# The influence of HIV on return to work in stroke survivors amongst a population of the eThekwini district, Kwazulu Natal: A qualitative study

**DOI:** 10.1177/10519815251323999

**Published:** 2025-04-17

**Authors:** Wesley Stephen Naidoo, Nicolette Comley-White, Mokgobadibe Veronica Ntsiea

**Affiliations:** 1 Department of Physiotherapy, School of Therapeutic Sciences, University of the Witwatersrand, Johannesburg, South Africa

**Keywords:** antiretroviral therapy, unemployment, stakeholder participation, economic productivity, cerebrovascular accident, vocational rehabilitation, quality of life, health-related quality of life

## Abstract

**Background:**

Biomedical and biopsychosocial factors with an interaction of systems and stakeholders are considered determinants of participation in a return to work (RTW) process. Human Immunodeficiency Virus (HIV) potentiates the risk of stroke development and employees that may be productive are negatively impacted economically from resultant disability. Evidence on the influence of HIV on the RTW of stroke survivors is scarce.

**Objective:**

To explore the influence of HIV in stroke survivors who either have or have not RTW.

**Methods:**

A qualitative study with a phenomenological approach using semi-structured interviews was conducted. Twelve HIV positive stroke survivors with a history of employment prior to stroke onset, were recruited from an HIV clinic database.

**Results:**

Stroke related physical and cognitive impairments impacted activities of daily living and occupational tasks. Health education aided participants to accept their HIV status and antiretroviral therapy (ART) adherence which promoted longevity amidst acute side effects. During the RTW effort, disclosing the HIV status, eliminating societal barriers, and having a family support structure were considered factors that might increase the desire for continuous employment and restore an acceptable quality of life.

Workplace support structure, realistic goal setting, engagement with RTW-related professionals and support services, alleviation of negative employer attitudes and avoidance of undesirable workplace accommodation were noted to promote economic productivity.

**Conclusion:**

Disclosing both HIV status and ART usage encourages adherence to medical care and explains subsequent absence from work. Full disclosure could result in understanding from the employer and resultant positive economic productivity.

## Introduction

People living with HIV (PLHIV) are predisposed to the development of stroke.^1^ A stroke excludes neurological deficits from epidural and subdural haemorrhage, trauma, malignancy and infection-related cerebral haemorrhage or infarction, and transient ischaemic attacks characterized by “brief episodes of focal loss of brain function of less than 24 h and with no persistent deficit”.^2^ Atypical stroke presentations are common in the context of HIV infection—e.g., acute confusion, fever, acute loss of consciousness, and stepwise focal neurological presentation over hours to days.^3–5^ Stroke is one of the most common and resource intensive diseases.^6,7^ There are increasing incidental rates of stroke in low- and middle income countries where HIV is often prevalent,^1^ with over two-thirds of the world's HIV population found in sub-Saharan Africa.^8^ With 7.9 million people estimated to be living with HIV in South Africa,^9^ 26.4% are adults aged 25–49 years old and 1.43 million of those PLHIV are found in Kwazulu-Natal.^10^ Without considering common risk factors such as diabetes mellitus, hypertension, hypercholesteremia, excessive alcohol intake, inactivity, and smoking,^11^ the mean age of PLHIV, found across different regions globally, presenting with a stroke is 40 years.^12–17^ As working-age individuals (defined as an individual between 18 and 64 years old)^18^ are part of an economically active population,^19^ the increased risk of stroke amongst workable or employable adults poses a serious threat to their livelihoods.^20^ The workplace serves as an important location of economic activity, upon which individuals depend for social and individual development, and often for their very survival.^21^ The perceived effects of illness and chronic diseases as well as the associated treatments in physical, emotional and social wellbeing (known as the health-related quality of life (HRQoL))^22–24^ requires exploration to construct a return to work (RTW) strategy for a person removed from their occupation, which is considered a life role.^25^ Socioeconomic status and the need to survive forces stroke survivors in sub-Saharan countries to continue to work beyond the age of 65 years old,^25^ even though their mean age is lower than sixty years of age.^26^

For young stroke survivors, resuming work indicates recovery^27^ with a significant impact on subjective well-being and life satisfaction.^28^ Returning to work is seen as a positive influence on QoL amongst stroke survivors in a South African based study^29^ which, by another South African based study, was affected by the level of physical functioning on functional outcomes and community reintegration.^30^

Predictors of RTW in stroke survivors are a greater independence in activities of daily living (ADL);^31–35^ better cognitive ability;^36,37^ fewer neurological deficits;^29,31,32,36^ and employment in an office or professional setting referred to as white collar type jobs.^32,35,38,39^ Amongst PLHIV, similarities can also be seen with a higher level of education safeguarding against unemployment^40,41^ as well as job type; and less likely to be employed with greater physical and psychiatric health challenges.^42,43^ Symptoms of HIV, co-morbidities and unemployment are part of a consistently reported list of adverse effects on HRQoL.^44^ The advent of antiretroviral therapy (ART) and early initiation to effectively manage PLHIV^45^ means they can survive longer and grow older but increases the likelihood of age-related diseases such as stroke.^46–48^ ART, whether initiated in the early or late stages of the disease progression, allows PLHIV to maintain their employment.^49,50^ The latter stages of ART initiation, however, saw a shift in returning to less stable and less profitable employment than their previous employment or work engagements.^51^ South Africa, which bears a high HIV burden, battles multimorbidity (defined as multiple medical conditions occurring simultaneously in a single individual)^52^ as it is occurring at younger ages due to their elevated levels of cholesterol and triglycerides.^53^ Multimorbidity was analysed in a large sample of adults in rural Kwazulu-Natal and regardless of disease control, the presence of non-communicable diseases (stroke included) was associated with a poor HRQoL. The success of ART in reducing HIV virulence makes the disclosure of one's HIV diagnosis less urgent, as is its influence on the ability to work.^54^ HIV status disclosure is a complex issue, with a considerable impact on the QoL of PLHIV.^55^ HIV-related discriminatory practices experienced at the workplace increased the likelihood of, or eventual unemployment/.^56^ If work-related health concerns are intensified due to disability or unemployment, PLHIV are less likely to consider RTW.^57^ They are more likely to commit to a RTW process if their health distress is reduced and health perception improved.^58^

A Cochrane systematic review identified four studies that found low quality evidence that ART interventions may improve employment outcomes for PLHIV however no intervention has been found to sustain and improve employment or successful RTW amongst PLHIV.^59^ An African study shows the benefit in RTW facilitations amongst stroke survivors^29^ but no research is available to support RTW initiatives and facilitations in HIV positive stroke survivors. The scarcity of evidence on the influence of return to work of stroke survivors who are HIV positive, lead to this study in a novel field.

## Methods

This was a qualitative study with a phenomenological approach^60,61^ using a demographic questionnaire and a semi-structured interview. Study participants were stroke survivors who were HIV positive, employed prior to their first incident of stroke and no less than six months post stroke. The researchers had no prior knowledge of, or interaction with, the participants. Following ethical approval (clearance certificate no. M170602) and permission obtained from the management at the regional hospital used, prospective participants were identified from a regional hospital's HIV clinic records in South Beach, Durban, Kwazulu-Natal. Prospective participants were excluded if they were pensioners, aphasic or of unknown HIV status. During telephonic tracing, prospective participants with incorrect contact details, out of the province at the time of study or voluntarily refusing to participate were excluded.

Potential participants were informed of the purpose of the study and the confidentiality of all information before written informed consent was obtained in their preferred language i.e., English or isiZulu. They also consented to the use of a digital voice recorder for the semi-structured interview.

A pilot study was conducted during June 2018, for the researchers to familiarize themselves with the demographic questionnaire and interview schedule. Minor adjustments to the demographic questionnaire and semi-structured interview were made accordingly. The demographic questionnaire was administered in participants’ preferred language i.e., English or isiZulu, with assistance rendered and completed by either the principal researcher or research assistant. It built a profile of the study population in respect to their personal information, related medical information, economic and employment status, type of work and mode of transport to work.

The main study was conducted from August 2018 until October 2018. Once the demographic questionnaire was completed, the semi-structured interview was audio-recorded, with participants detailing events leading up to and beyond their first incident of stroke as well as how stroke, HIV and ART adherence affected their life in a RTW process. Four participants requested that the interview be conducted in isiZulu to enable an expressive platform, which was led by the research assistant with the principal researcher present. The other eight participants were interviewed in English, which was led by the principal researcher. The semi-structured interview guided by the open-ended questions was used and is presented as Annexure A. The semi-structured interview began, being digitally voice recorded in a noise-reduced environment within the regional hospital, between the participant and the primary researcher face to face. All interviews started by asking the participant to talk about something important to them, thereafter with “the stroke onset” followed by “the HIV and ARV onset” and coherently bringing in the work aspect. The demographic questionnaire took an average of five minutes to complete, and the semi-structured interview lasted between 16–61 min.

Data collected from the demographic questionnaires was electronically recorded using the sub-headings of the profile into an electronic spreadsheet and analysed using frequencies and percentage distribution. The interviews were transcribed verbatim by an independently dedicated transcriber for consistency^62^ and conceptual congruency.^62,63^ Interviews conducted in English were transcribed verbatim. Interviews conducted in IsiZulu were translated and transcribed verbatim but needed to be logical and sensible to an English-speaking reader. Field notes and reflective journaling were done and shared by the principal researcher and research assistant for English and isiZulu-based interviews, respectively. An inductive thematic analysis of the transcripts from the interviews was done. The transcripts were read, with relevant statements highlighted, and data was clustered to develop codes. There was a constant comparison of all transcripts to ensure optimal and thorough coding.^64^ Data were independently coded by the researcher and at least one other independent individual (VN), including peer-debriefing and coding consistency checking by two other independent individuals (SC and KS) who ensured the credibility of the analysis. The independent reviewers had experience and knowledge of primarily qualitative research analysis. The relevant quotations were attached to the codes and documented into spreadsheets of an electronic workbook. When coding was complete, categories and sub-categories were developed and interconnected to understand relevant contributions, forming eventual themes. This was documented in an electronic worksheet.

## Results

Data saturation was reached with 12 participants interviewed. Their mean age was 39.2 years (range 29–54). The sociodemographic and clinical characteristics of the study participants are presented in Table 1. Ten of the participants were financially involved in their households when diagnosed with stroke, five of them as sole breadwinners. Only two participants held a form of employment, with four participants having attempted to RTW. All study participants were initiated on combined ART. Information about the participants’ type of work and mode of transport used to work is presented in Table 2. Eight participants engaged in work that involved physically demanding tasks whilst the remaining four participants (A, E, J and L) engaged in less physically demanding work. Eight participants used public transport to work.

**Table 1.** Sociodemographic and clinical characteristics of the study participants (N = 12).

Sociodemographic information:	
*Age (Standard deviation):*	39.2 years (7.85)
*Gender:*	
Male/ Female	6 / 6 (50/50)
*Race:*	
Black	12 (100)
*Education:*	
Intermediate Phase	1 (8.3)
Senior Phase	1 (8.3)
Further Education and Training Phase	8 (66.6)
Tertiary Level	2 (16.6)
*Residence:*	
Owned	2 (16.6)
Rented	2 (16.6)
Shared with family/ friends	8 (66.6)
*Financial role when diagnosed with HIV:*	
Sole breadwinner	7 (58.1)
Breadwinner/ contributing	4 (33.2)
Dependent	1 (8.3)
*Financial role when diagnosed with stroke:*	
Sole breadwinner	5 (41.5)
Breadwinner/ contributing	5 (41.5)
Dependent	2 (16.6)
*Receiving a Disability grant:*	
Yes	4 (33.2)
No	8 (66.6)
*If No, have they applied to receive a DG?*	3 (24.9)
Yes	4 (33.2)
No	1 (8.3)
Pending	
*Received any benefit from private insurance?*	
Yes	2 (16.6)
No	7 (58.1)
Never heard of such	3 (24.9)
*Participant currently working?*	
Yes	2 (16.6)
No	10 (83.3)
*Participant attempted returning to previous job?*	
Yes	4 (33.3)
No	8 (66.6)
*Did anyone help them understand being accommodated RTW?*	
Yes	4 (33.3)
No	8 (66.6)
*If Yes, who was it?*	
Healthcare workers	4 (33.3)
*Preference of employment status:*	
Continue to work	6 (50)
Seek another job	6 (50)

Clinical characteristics	
*On Antiretroviral treatment (ART):*	
Yes	12 (100)
*Weaker side of body:*	
Left	9 (74.7)
Right	3 (24.9)
*Dominant side of body:*	
Left	2 (16.6)
Right	10 (83.3)
*Receiving treatment following stroke from:*	
Medical doctor	10 (83.3)
Physiotherapist	11 (91.6)
Occupational Therapist	3 (24.9)
Speech and Language Pathologist	1 (8.3)
Audiologist	1 (8.3)
Social Worker	3 (24.9)
Dietician	2 (16.6)
Counsellor	2 (16.6)
Traditional Healer	2 (16.6)
*Medical conditions known about:*	
Hypertension	3 (24.9)
Hepatic conditions	2 (16.6)
Asthma	1 (8.3)

**Table 2.** Participants’ type of work and mode of transport used to work.

Participant	Job Title	Job Description	Mode of Transport to work
A	Kitchen assistant	Cooking various dishes	Private as passenger
B	**Customer service agent**	Attending to network and client-related queries	Public
C	General cleaner	Mopped and dusted (shop keeping)	Public
D	Machine operator	Planned and manufactured radiators	Public
E	**Recruitment agency supervisor**	Administrative tasks including paymaster duties	Public and private
F	Domestic worker	Housekeeping duties and aftercare of children	Stayed at workplace
G	Enrolled nursing assistant	Medical related care for patients in hospital	Public
H	Combi conductor	Co-ordinating trips and collecting fares	Public
I	Construction worker	Transporting mixed mortar and bricks	Private as passenger
J	**Front desk receptionist**	Recorded occupancy and payments at lodge	Stayed at workplace
K	Pool pump installer	All activities involved in installing home-built pools	Public
L	**Financial advisor**	Undertaking and underwriting insurance policies	Public and private

The participants were asked about events leading up to and beyond their first-ever stroke incident as well as how stroke, HIV and ART adherence affected their life. Developed themes, categories and sub-categories related to the effects of stroke sequelae on various life roles and relevance to HIV status in RTW scenario are presented in Table 3 and elaborated on below.

**Table 3.** Themes, categories and subcategories developed from interviews of the study participants.

Themes	Categories	Subcategories
1. The effects of stroke sequelae on various life roles	Physical impact Cognitive impact	Ability to use transport. Independence in Activities of Daily Living (ADL) performance. The effects of stroke sequelae causing upper limb impairments to ADLs. The effects of stroke sequelae causing lower limb impairments to ADLs. The effects of physical stroke sequelae on occupational tasks. The effect of cognitive stroke sequalae on occupational tasks.
2. Relevance of HIV status in RTW scenario	Psychotherapy and counselling Engagement with RTW professionals	Fear created by lack of knowledge regarding: the HIV threat to life and continued employment following HIV disclosure Time off to continue treatment
3. ART considerations	Fear of adverse effects Promoting longevity of life with continued ART	Continued ART adherence. Defaulting ART regime. A lifeline towards a continued livelihood.
4. Economic factors	Positive economic productivity Negative economic productivity	To maintain employment. Restore prior employment benefits Employer's negative attitude. Lack of support during RTW process. Undesirable workplace accommodation.
5. Societal factors	Barrier to an acceptable QoL Family support	Social stigma. Family support structure towards RTW efforts.
6. Future QoL and RTW prospects	Promoting a better QoL Realistic goal setting towards new job opportunities	An improved outlook through employment. Impact of altered health status on work stability. Benefits of returning to economic productivity.

### Theme 3.1: The effects of stroke sequelae on various life roles

Each participant individualized their diverse physical sequelae, with fewer participants discussing their cognitive sequelae, following their respective stroke incident.

“My life is not the same as before now, sometimes I think that maybe it would have been better if the stroke would have just killed me, because now the quality of life has deteriorated, I sometimes have the feeling of just wanting to be isolated and stay alone.” Participant H

The resultant stroke sequelae affected their activity and participation levels towards transport and ADL performance.

“The biggest challenge was boarding a taxi. I had to be carried so that I could get on.” Participant E

“I could not walk. I had to stop working. I had to stop even running. I used to run. I used to play soccer. I stopped working. I stopped to support my child because now I am not working.” Participant G

Following their respective stroke incidents, participants evaluated their residual abilities towards their current occupation. There was an overwhelming fear that due to the resultant sequelae, they doubted their productivity levels which would compromise their workplace performances.

“My work relied heavily on me using my hands, feet and applying my mind. I often made use of a ruler, a measuring tape, a ball pen and I was required to think all the time. So, I had to think about my wellbeing. I could not operate a press with my feet, and I could not operate the machine with my hands because the stroke had an impact on their functioning.” Participant D

“Because I can’t walk, and I can’t talk properly… That is the reason. I can’t talk. My job was to interact with the client, so now I can’t talk… I understand the advisors work so you see my office there's no admin. There's no work that will make you sit down and do. I have to go out and make money.” Participant L

### Theme 3.2: Relevance of HIV status in RTW scenario

Psychotherapy and counseling enabled the participants’ acceptance of their HIV status. Most of their responses on their experience of a RTW process illustrated their desire to be valued as part of the workforce. A disclosure of their HIV status illustrated their commitment to remain employed. They felt that forging a collaborative relationship and being accommodated at the workplace would allow them to meet their medical needs and potentially engage with RTW enabling services.

“So, I’ve been exposed to family with HIV and AIDS I kind of knew about it even though I thought it was far from me because of the behaviour that I had, even now I still can’t believe that I have this kind of disease…I managed to get over it because I attended a lot of what is called? Psychologist, counselling. They even referred me here and I’ve been attending up until I felt like I’m fine now.” Participant B

“They really need to know that on a certain date I will not be coming to work because I must collect my medication. He will at least know where I am instead of assuming that I am absent from work.” Participant A

“After I worked with a physio. I picked up and started walking. Yes, it was for a couple of months.” Participant E.

### Theme 3.3: ART considerations

Some participants faced a dilemma between ongoing and defaulting ART adherence.

“At first it changed my life because at some point I was too big (gained weight) and I was confused and it was like I was a fool, and then things went back to normal. ”Participant F

“Following year, I got tired .it wasn’t even a year, and I felt like I don’t want that medication anymore and then I stopped. I didn’t go to get medication for about 3 months. I wasn’t sick because even at that time they were telling me I will get sick, I wasn’t sick. I was still fine. I didn’t want to take it but now I can see that it is helping me a lot because the time I was here in hospital they told me that the CD4 count is over 1000.” Participant B

The chosen path explored brought initial doubt, subsequent adverse effects with a few cases ending in hospitalization but eventually promoted the idea of treatment continuity. Improvement to their overall health was encouraged by treatment continuity which renewed the focus on a continued livelihood despite not returning to their former livelihood.

“I won’t say it's the same but I’m alive, but I can say it has put it back to normal. I’m not that weak now, I take my pills accordingly, even though it will not go back to the way it was before (my life), but they are helping me.” Participant I

“All I can do is focus on my treatment and make sure that it does not progress further because I cannot stop it. Therefore, there is life if you adhere to treatment.” Participant D

### Theme 3.4: Economic factors

The idea of economic productivity was established. Participants highlighted that to attain positive economic productivity, they should sustain their employment. The following participant's response speaks to the ability of a workplace accommodating their absence or time off:

“I think it's important because he needs to understand that when you are absent, he is aware about your reason to be absent. Your reason to be absent so he can make arrangements for your replacement and work smooth the next day.” Participant H

Conversely, participants shared their thoughts of employers’ fears manifesting should a negative economic productivity level at the workplace loom. If affected employees, post illness, lack a support system and workplace accommodation whilst engaging in a RTW process, it may be perceived as an undesirable workplace to return to.

“They were going to fire me. Because I heard that the time, I was in hospital they wanted to fire me, because of the stroke.” Participant J

“I worked at that company for about thirteen to fifteen years. To be honest, the employer only likes you while you are still fit to perform your duties… You must push his production so that he can get what he wants from you. He is not worrying about your life.” Participant D

Participants felt that they can reserve the right to disclose their health status, particularly their HIV status, to the employer as it had no relevance to the productivity level of their work performance. The need to have a consultative, collaborative RTW process with the affected employee and the relevant workplace stakeholders, was emphasized as a priority.

“No, I don’t think it has anything to do with your work… The status has nothing to do with who you are and what you do.” Participant L

“They just came to me that your medical boarding has been approved. But they never came and try to analyse exactly what is it and how they can assist… and you know what I was turned down by this story that you can never work for the … since you took medical boarding, or you can never work in this section.”

### Theme 3.5: Societal factors

The loss or compromise to a person's level of independence, acquired through their livelihood, can be difficult to cope with. Some participants reported that they had been discriminated against because of their altered health status.

“It's difficult because when you are sick there's that stigma…They speculate that it's because of a certain disease or condition, but I eventually accepted.” Participant I

Societal support encouraged participants to persevere in returning to economic productivity even though it was their stroke sequelae that burdened them. An example of societal support is illustrated by family intervention which comprised of financial, moral, caregiving and improved quality of life support. This can encourage the thoughts of, and ease the engagement towards, a new job.

“Some say I must study to be a cashier. That course is very expensive. Some are negative. They say I will not work anywhere because I use one hand.” Participant A

“We have a family business. They knew what I was doing before. So, we had difficulties in our business. They took me and said I do that, and now the business is doing very well.” Participant E

### Theme 3.6: Future QoL and RTW prospects

The prospect of entering a new job setting was seen as being a tool for health promotion towards an improved QoL.

“Where I am right now, it helps me a lot because I can do some exercising instead of sitting at home and doing nothing. It helped me a lot.” Participant E

A change in the job setting improved the participants’ perceived prognosis. It revived a return to economic productivity that would improve their socioeconomic state, guided by the realistic goal setting towards the suitability of work sought.

“I think I would be happy for a new job, and it will improve my life even at home… supporting my family at the most. Because now I’m depending on my uncles who have better jobs at the moment and the kids need transport money to school and a lot of other things.” Participant I

“Every job out there is designed for me, I think. Unless if it is a job that requires physical strength. Every type of work suits me, I can do it. If they ask me to manufacture a car, I will tell them what I need to make that car. That job I can do as well. I can even work at a call centre. I can talk to people.” Participant D

## Discussion

This study investigated the influence of HIV on stroke survivors’ RTW in a population of the eThekwini district, Kwazulu-Natal. It described the demographics and clinical characteristics, established the rate of RTW and gained insight into the reasons for either RTW or not of stroke survivors who are HIV positive. Studying the experiences and meaning of a disease aided in exploring personal and sensitive themes. Such exploration can help identify potentially modifiable factors to improve health care.^65^

The mean age of this population was like HIV positive stroke survivors studied in previous works.^11,12,17,18,66^ Like a study by Heikinheimo,^11^ only 25% (N = 12, n = 3) of the study population developed a stroke within a year. A possibility is the effect of immune reconstitution inflammatory syndrome and associated vascular endothelial pathological changes, but this assumption requires further research. With the remaining population developing a stroke beyond a year post ART initiation, regular medical screening of PLHIV to evaluate the risk of stroke development or other side effects may be considered.

At least 90% (N = 12, n = 11) of participants had their breadwinner statuses compromised following the sudden loss of employment due to their altered health status, whether it was the stroke onset or HIV diagnosis. Most participants worked in small or medium business enterprises that were potentially unable to afford the provision of disability insurance to their employees. Only a third of the study participants and two others earned a state dependent disability grant or private disability benefit, respectively. No literature was found on stroke survivors’ accessibility to disability grants in South Africa, but PLHIV are amongst those vulnerable populations that receive state aided disability grants to relieve poverty and protect economic shock.^67^ Introducing affordable disability insurances at the workplace and uniform, formal procedures in respect to accessibility of disability grant applications would further relieve and protect vulnerable populations^68^ including HIV positive stroke survivors. During recovery in a RTW process (with considerations that included alternative job seeking), the provision of economic support to maintain an acceptable QoL and HRQoL may alleviate the affected employee's stress and burden on society.^69^

The need for vocational rehabilitation is advocated amongst affected employees whose work suitability is precipitated by their altered health status. Attributed to the greater number of blue-collar occupations amongst studied participants, there was a heightened emphasis placed on the physical impairments following the stroke event. Only two participants had tertiary level education though a definitive achievement was not ascertained. A higher education level, clearly defined career path and work experience can favour the likelihood of an earlier RTW through appropriate vocational rehabilitation.^70^ Work trials provide important information and insight into the RTW process. There must be a comprehensive length of time to evaluate the success of a work trial towards a former or new job post. In the studied population, their decision to RTW in a former or new job post and whether they wanted a restoration of employment benefits or breadwinner status was brought into question. Due to the multitude of effects on various life roles due to stroke,^6,7^ it is a resourcefully demanding condition to work with. A graded RTW approach with the inclusion of work adaptations or possibly entering different job types can enhance the RTW initiative and work sustainability following a stroke.^64,71,72^ As with PLHIV that increased their level of work consideration by reducing health distress^58^ stroke survivors similarly would benefit. A consultative RTW process could have mitigated the premature decision to RTW as fears of further socioeconomic crisis troubled a few of the studied participants.

The desire to seek a new job opportunity does not guarantee a restoration of prior employment benefits, but from this studied population, it may be a search for work to minimize the negative effect on their QoL. It is well documented that in both PLHIV and stroke survivors, a person's highest level of QoL and HRQoL illuminated the viability and realism of work. A greater independence in ADLs may promote the higher level of QoL and functioning to avoid work related disability.^73^ An overall support system may improve the outcomes for a better QoL. Due to the lack of social support, two participants had struggled to navigate a consultative RTW process, both sighting that their social well-being was compromised. The lack of social and family support^73,74^ resulted in their work contemplation therefore participants viewed a holistic support system as a culminating factor to the restoration of a breadwinner status.

Apart from the exclusion of HIV positive aphasic stroke survivors, we can only speculate on the recruitment of other services including healthcare in holistically managing stroke survivors who are HIV positive. Medical doctors and physiotherapists were mostly consulted, owing to the obligatory follow-up appointments on patient prognosis as primary healthcare providers during the patient's stroke onset.^75^ The interaction of multi-morbidities on stroke survivors who are HIV positive returning to work was not a part of the study's objectives. We had noted a low representation or reporting of only three co-morbidities i.e., Asthma, Hepatic-related conditions and Hypertension. There were no responses of such interaction with any of the participant's resultant stroke, HIV or ART in in their RTW effort. With a greater sample size, the exploration of multi-morbidity and its influence on RTW efforts in this interest group would likely empower RTW enabling healthcare and non-healthcare professionals to enrich pivotal inter-referral patterns that can lead to more meaningful stakeholder intervention and cooperative initiatives. A consultative RTW process may have better informed an equal ratio of studied participants’ consideration to RTW to their former job compared to those seeking a new job as only a few were guided frankly by their healthcare professionals on a RTW process.

The various stroke sequelae that challenged studied participants, disclosing their HIV status as well as comply with their ART regimes unprejudiced at the workplace to maintain a positive, economically productive work environment challenged their job security. A few of the participants viewed disclosure of their HIV status as irrelevant to their job performance but were fearful of workplace discrimination which is considered a barrier for PLHIV to RTW.^59,76^ Participants acknowledged that their ART adherence brought a positive effect on RTW productivity which enabled a disclosure of their HIV status. A continuous ART adherence despite the presence of residual stroke sequelae can potentially prevent side effects evolving that may lead to unforeseen medical consultations, hospital admissions and an unfavorable QoL that prolongs time away from work engagement. The knowledge of an affected employee's medical needs will assist in collaboration with employers to plan for absence from work, which can contribute to stimulating a positive, economically productive work environment. Evidence from Sub-Saharan African research showed that ART adherence from as early as a month post HIV diagnosis resulted in a decrease of absenteeism and conversely an increase in work productivity and performance.^77^ Introducing a wholesome and ongoing health education program with an involvement of all stakeholders in the workplace can harness the positive, economically productive work environment.^66^ Incentivized health awareness initiatives where possible, such as continuous in-service training sessions; guest and motivational speakers and work support groups towards employment wellness programs can aid to prevent prejudicial and stereotypical behaviour found in HIV affected populations at the workplace.^76,78^ It allows affected employees to disclose both their HIV status and ART regimes to encourage better workplace accommodation as well as maintain employment.^59^ These initiatives may need to continue after an affected employee returns to work as hidden problems could only appear several months post RTW.^79^

## Conclusion

An integral component for a stroke survivor who is HIV positive, besides being able to resume work is for the workplace to accommodate them and accomplish their previous work tasks to satisfy the work demands. This study illustrated the consequences suffered by a group of stroke survivors who were HIV positive. Their altered health status warranted the consideration for continued versus productive employment that would restore or maintain an acceptable socioeconomic status through vocational rehabilitation in a consultative RTW process. Their health related, work related, and societal support factors influenced their decisions and outcomes that followed. A shift towards health education to alleviate discrimination and stigmatization may culminate a collaborative RTW process between all stakeholders including the affected employee at the workplace. A simultaneous disclosure of their HIV status and use of ART would substantiate their ongoing need for medical care hence time away from work and empower employers to maintain a positive, economically productive work environment during their absence. Having a support structure and maintaining employment post HIV and ART disclosure stimulates positive economical productivity. RTW enabling professionals will inform better decision-making and stimulate realistic goal setting towards a return to a former or new job opportunity.

The definition of RTW^25,80^ used in this study (of returning to a work environment) for the recruitment of participants employed prior to their first incident of stroke may have limited the exploration of self-employment. HIV positive stroke survivors that can attain entrepreneurial knowledge and skills as a means towards new job opportunities being sought may further inform the concept of economic productivity. This can be included into possible longitudinal studies investigating the contribution of RTW-enabling professionals and other support services towards a restorable QoL as a potential outcome measure. The data saturation achieved from this homogenous sample of participants illustrated a fair representation of thoughts and experiences. Further research into this unique population should include a snowball sampling strategy to include a greater recruitment of white collared occupations to explore further challenges, not yet known, in their workplace participation or RTW process. Although no other literature was found exploring the RTW process of stroke survivors who are HIV positive, other relevant research will inform and improve perspectives to advocate for an improved universal RTW approach.

## Annexure A: Interview questions

1.
  - You had the stroke in (mention from questionnaire). Tell us what happened?
  - Can you remember what difficulties you had before the stroke?
  - Can you remember what difficulties you had after the stroke?
  - How has the stroke affected your life?
  - How has HIV affected your life?
  - **(If on treatment)** How has the ARV treatment affected your life?
2.
  - Briefly, describe your work.
  - How far was it from your home to work? How did you travel to work and home?
  - Why/why not have you returned to work?
  - Do you think HIV, stroke or both brought you to this point? Why?
  - What challenges have you encountered RTW? How have these been addressed?
  - How did your employer accommodate your RTW? [lighter/alternate work, asking them, their responses]
  - Who else at your workplace assisted in trying to accommodate or help you through your difficult times or return to work or finding a new job? Tell us what did they do for you?
  - Who else in your life assisted in trying to accommodate or help you through your difficult times or return to work or finding a new job? Tell us what did they do for you?
  - Did you reveal your status to your employer? Why/ Why not?
  - Is it important to reveal your status to your employer? Why?
  - How do you feel about returning to work? Why?
  - How do you feel about finding a new job? Why?
  - Is there a job available for you? If NO, why? If YES, what job do you think is available for you and why?
  - What do you believe are factors that could facilitate and promote someone similar RTW? Why?
  - What do you believe are factors that could be obstacles that affect someone similar RTW? Why?
3. Is there anything, related to your stroke and returning to work, that you want to talk about?
